# LncRNA *MORT (ZNF667-AS1)* in Cancer—Is There a Possible Role in Gynecological Malignancies?

**DOI:** 10.3390/ijms22157829

**Published:** 2021-07-22

**Authors:** Riccardo Di Fiore, Sherif Suleiman, Rosa Drago-Ferrante, Ana Felix, Sharon A. O’Toole, John J. O’Leary, Mark P. Ward, James Beirne, Angel Yordanov, Mariela Vasileva-Slaveva, Yashwanth Subbannayya, Francesca Pentimalli, Antonio Giordano, Jean Calleja-Agius

**Affiliations:** 1 Department of Anatomy, Faculty of Medicine and Surgery, University of Malta, MSD 2080 Msida, Malta; sherif.s.suleiman@um.edu.mt; 2 Sbarro Institute for Cancer Research and Molecular Medicine, Center for Biotechnology, College of Science and Technology, Temple University, Philadelphia, PA 19122, USA; president@shro.org; 3 BioDNA Laboratories, Malta Life Sciences Park, SGN 3000 San Gwann, Malta; rosa.dragoferrante@biodna.net; 4 Department of Pathology, Instituto Portugues de Oncologia de Lisboa, NOVA Medical School, University NOVA of Lisbon, Campo dos Mártires da Pátria, 130, 1169-056 Lisbon, Portugal; ana.felix@nms.unl.pt; 5 Departments of Obstetrics and Gynaecology, Trinity St James’s Cancer Institute, Trinity College Dublin, D08 HD53 Dublin, Ireland; shotoole@tcd.ie; 6 Department of Histopathology, Trinity St James’s Cancer Institute, Emer Casey Molecular Pathology Laboratory, Trinity College Dublin and Coombe Women’s and Infants University Hospital, D08 RX0X Dublin, Ireland; olearyjj@tcd.ie (J.J.O.); wardm6@tcd.ie (M.P.W.); 7 Department of Gynaecological Oncology, Trinity St James’s Cancer Institute, St James Hospital, Trinity College Dublin, D08 X4RX Dublin, Ireland; JBeirne@stjames.ie; 8 Department of Gynecologic Oncology, Medical University Pleven, 5800 Pleven, Bulgaria; angel.jordanov@gmail.com; 9 Department of Breast Surgery, Acibadem City Clinic, 1750 Sofia, Bulgaria; sscvasileva@gmail.com; 10 Centre of Molecular Inflammation Research (CEMIR), Department of Clinical and Molecular Medicine (IKOM), Norwegian University of Science and Technology, 7491 Trondheim, Norway; yashwanth.subbannayya@ntnu.no; 11 Cell Biology and Biotherapy Unit, Istituto Nazionale Tumori-IRCCS-Fondazione G. Pascale, 80131 Napoli, Italy; f.pentimalli@istitutotumori.na.it; 12 Department of Medical Biotechnologies, University of Siena, 53100 Siena, Italy

**Keywords:** *MORT* (*ZNF667-AS1*), long non-coding RNA, epigenetics, ovarian cancer, cervical cancer, endometrial cancer, epigenetic therapy, lncRNA-based therapy

## Abstract

Gynecological cancers (GCs) are currently among the major threats to female health. Moreover, there are different histologic subtypes of these cancers, which are defined as ‘rare’ due to an annual incidence of <6 per 100,000 women. The majority of these tend to be associated with a poor prognosis. Long non-coding RNAs (lncRNAs) play a critical role in the normal development of organisms as well as in tumorigenesis. LncRNAs can be classified into tumor suppressor genes or oncogenes, depending on their function within the cellular context and the signaling pathways in which they are involved. These regulatory RNAs are potential therapeutic targets for cancer due to their tissue and tumor specificity. However, there still needs to be a deeper understanding of the mechanisms by which lncRNAs are involved in the regulation of numerous biological functions in humans, both in normal health and disease. The lncRNA *Mortal Obligate RNA Transcript* (*MORT*; alias *ZNF667-AS1*) has been identified as a tumor-related lncRNA. *ZNF667-AS1* gene, located in the human chromosome region 19q13.43, has been shown to be silenced by DNA hypermethylation in several cancers. In this review, we report on the biological functions of *ZNF667-AS1* from recent studies and describe the regulatory functions of *ZNF667-AS1* in human disease, including cancer. Furthermore, we discuss the emerging insights into the potential role of *ZNF667-AS1* as a biomarker and novel therapeutic target in cancer, including GCs (ovarian, cervical, and endometrial cancers).

## 1. Introduction

Gynecological cancers (GCs) arise from the female reproductive organs and include tubo-ovarian, cervical, uterine, vaginal, and vulvar cancers. One of the main risk factors is the age at presentation, with increasing age being directly related to increased risk of developing GCs [1]. Even though signs and symptoms may overlap, each GC may present with different risk factors and might require different therapeutic approaches [2,3]. The mortality rate among patients with GCs is high because many cancers are detected at an advanced stage [1].

Histologically, different types of tubo-ovarian, cervical, and endometrial cancers have distinct pathological behavioral patterns that place these tumors into a ‘rare’ category, with an annual incidence of <6 per 100,000 women [4]. Rare gynecological cancers (RGCs) represent over 50% of all gynecologic tumors, which is in contrast with other common solid tumors [5]. RGCs tend to be associated with poor prognosis, and given their rarity and, thus, low incidence in the case of each respective entity, there is a greater risk of delayed diagnosis due to clinical inexperience and the lack of availability of therapeutic options. These are a few of the main challenges in the management of patients with RGCs [5,6,7,8]. Another major hurdle in developing standardized clinical management guidance is the fact that the treatment of RGCs tends to be based on expert opinion, retrospective studies, or extrapolation from other tumor sites with similar histology [5,6]. 

Despite many studies contributing to understanding disease pathophysiology, research in the field of GCs poses particular scientific and technological difficulties. As such, these challenges require a concerted effort by the scientific and research communities to accelerate the advancement of prognostic, diagnostic, and therapeutic approaches [6]. Human transcriptomic analyses, by DNA tiling arrays and deep sequencing, have shown that only a minor portion of the genome is transcribed into mRNA, while the vast majority constitutes non-coding RNA (ncRNA) [9,10,11,12]. These ncRNAs are further subdivided into small ncRNA and long ncRNA (lncRNA), based on the nucleotide length, with small ncRNA containing less than 200 nucleotides and lncRNAs exceeding the 200 nucleotides threshold. Furthermore, lncRNAs are transcribed in a similar fashion to the messenger RNA (mRNA) using RNA polymerase II with 5′ end-capping, polyadenylation of the 3′ end tail, and ultimately splicing. Most lncRNAs are likely to be functionally important because they are strictly regulated and evolutionarily conserved [13]. Many studies have shown that lncRNAs have an important role in gene expression. Some short open reading frames of lncRNAs have been shown to encode into micropeptides, which can modify N6-methyladenosine, tumor angiogenesis, cancer metabolism, and signal transduction, and thus have a potential clinical value [14]. Functional studies reveal that the influence on key genes occurs via various mechanisms, such as chromatin modification, transcriptional, and post-transcriptional regulation [15,16]. Furthermore, lncRNAs interact with microRNAs (miRNAs/miRs), mRNAs, proteins, and genomic DNA to bring about physiological and, at times, pathological actions [17,18]. In tumorigenesis, lncRNAs can behave both as tumor suppressors or oncogenes, resulting in up- or downregulation of specific lncRNAs in comparison to corresponding normal tissues [17]. LncRNAs also play regulatory roles in cancer-related pathways, such as the Hedgehog, Wnt, Notch, and PI3K/AKT/mTOR pathways, and regulate the plasticity of cancer stem cells [17]. Dysregulation of several lncRNAs has been implicated in different types of cancers, include breast, ovarian, cervical, and prostate cancer. This suggests that lncRNAs could be fundamental as biomarkers for detection of cancer and monitoring prognosis as well therapeutic targets for cancer management [19].

The aim of this review article is to provide an updated overview of the emerging role of *mortal obligate RNA transcript* (*MORT*; alias ZNF667 antisense RNA 1 (*ZNF667-AS1*)), not only as a potential biomarker but also as a therapeutic target for novel oncologic treatments, including management of GCs.

## 2. LncRNA *MORT* (*ZNF667-AS1*): From Discovery to Function

**Figure 1:**
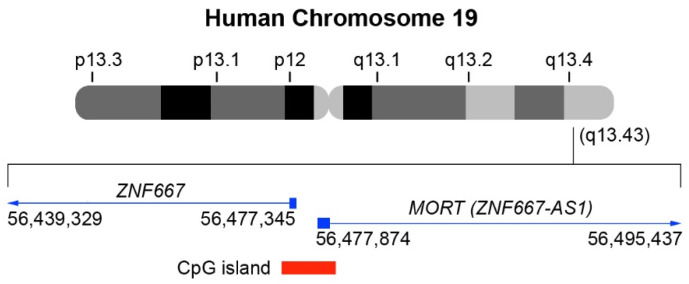
*MORT* (*ZNF667-AS1*) and *ZNF667* genomic location. *MORT* (*ZNF667-AS1*) and *ZNF667* are head-to-head antisense-sense strands. *MORT* (*ZNF667-AS1*) is also located in 19q13.43 (GRCh38p13 database, chr19: 56,477,874-56,495,437; NCBI: NR_036521.1). *ZNF667* is also located in 19q13.43 (GRCh38p13 database, chr19: 56,439,329-56,477,345; NCBI: NM_022103.4).

In humans, the *ZNF667-AS1* gene is located in the chromosome region 19q13.43 (Figure 1), at position 56,477,874–56,495,437 (Assembly GRCh38.p13) within a cluster of zinc finger genes [20]. Its 1.53 kb RNA consists of a 16 kb intron in between 2 exons of 260 and 1270 bp. The majority of the second *ZNF667-AS1* exon consists of repetitive elements—two LINEs (L2 and L1MB3) and an LTR (LTR47B) element [20]. Even though the *ZNF667-AS1* promoter is in a CpG island, which is shared with the non-homologous *ZNF667* gene, *ZNF667-AS1* and *ZNF667* genes do not overlap [20].

The molecular function of *ZNF667-AS1* remains poorly understood; however, *ZNF667-AS1* is preferentially expressed in the cell cytoplasm and may be involved in the regulation of protein translation via interaction with RNA-binding proteins [20]. Vrba et al. have shown that *ZNF667-AS1* silencing occurs prior to malignant transformation [20]. The study also demonstrates that *ZNF667-AS1* is ubiquitously expressed in normal healthy cells, but is lost when the cells are immortalized, and that *ZNF667-AS1* gene silencing occurs following DNA hypermethylation of its promoter. Overall, these findings suggest that *ZNF667-AS1* has a potential tumor-suppressive mechanism of action. 

## 3. The Functional Role of LncRNA *MORT* (*ZNF667-AS1*) in Human Diseases

*ZNF667-AS1* has also been associated with the pathogenesis of several other human diseases. It has been observed that *ZNF667-AS1* inhibits inflammation following spinal cord injury and, in turn, promotes faster recovery via the suppression of the Janus kinase-signal transducer and activator of transcription (JAK-STAT) pathway [21]. This pathway is involved in several key biological processes, including cell differentiation, proliferation, immune regulation, and apoptosis [21]. A deep transcriptomic profiling of macroscopically normal human Achilles’ tendon samples from young and old patients revealed a significantly reduced expression of *ZNF667-AS1* in older subjects. Changes observed in lncRNAs might be a contributing factor in age-related degeneration often seen in tendons [22]. A bioinformatics study using gene ontology and KEGG enrichment analysis of genes shows that, in asthmatic patients, the level of *ZNF667-AS1* is downregulated when compared to healthy controls. This suggests that it may play a role in the pathogenesis of asthma [23]. *ZNF667-AS1* was found to be upregulated in the plasma of patients with myocardial infarction when compared with healthy controls. *ZNF667-AS1* was found to increase cardiomyocyte cell death mediated by the downregulation of miR-93 [24]. Furthermore, high levels of *ZNF667-AS1* were found to correlate with a relatively lower recurrence rate of periodontitis. *ZNF667-AS1* was found to regulate the proliferation of periodontal ligamental stem cells isolated from periodontitis-affected teeth [25].

In cancer, *ZNF667-AS1* has been implicated in both promoting tumor progression and tumor suppression depending on the cancer type [26,27,28,29,30,31,32,33,34,35,36,37] (Table 1). In laryngeal squamous cell carcinoma (LSCC), decreased *ZNF667-AS1* levels were associated with increased migration and invasion together with increased levels of mesenchymal markers and a reduction in epithelial markers. These data suggest that part of *ZNF667-AS1′*s mechanism of action may lie in the epithelial-to-mesenchymal transition (EMT) process [26]. In oral squamous cell carcinoma, overexpression of *ZNF667-AS1* inhibits proliferation through the downregulation of Rho-associated coiled-coil containing protein kinase 1 (ROCK1) [27]. Chen et al. has shown that overexpression of *ZNF667-AS1* with subsequent binding of miRNA-1290 resulted in increased expression of the actin-binding LIM protein 1 (ABLIM1) and suppression of nasopharyngeal carcinoma (NPC) progression [28]. The fact that the downregulation of *ZNF667-AS1* exhibits an inhibitory effect on the viability, invasion, and migration of esophageal cancer cells suggests that it plays a tumor suppressor role in esophageal cancers [29]. Yang et al. investigated the role of *ZNF667-AS1* and its potential mechanism with MEGF10 [30], a type I transmembrane protein consisting of 17 EGF-like domains in the extracellular region, which has increased expression in the central nervous system, retina, myoblasts, and muscle satellite cells in tissues derived from uveal melanoma (UM). A reduced expression of *ZNF667-AS1* has been shown in UM tissues and its downregulation is correlated with poor prognosis, indicating that *ZNF667-AS1* may have an inhibitory role in the development of UM via the regulation of cellular aggressiveness [30]. Huang et al. investigated the role of *ZNF667-AS1* in lung adenocarcinoma (LUAD), where it was shown that *ZNF667-AS1* is downregulated, while miRNA-223 is upregulated [31]. Moreover, the expression of *ZNF667-AS1* is significantly affected by tumor metastasis, but not by the tumor size. The expression of *ZNF667-AS1* and miRNA-223 is also inversely correlated in LUAD with overexpression of *ZNF667-AS1* inhibiting the invasion and migration of LUAD cells, whereas overexpression of miRNA-223 has an opposite effect. These findings suggest that *ZNF667-AS1* may inhibit cancer cell migration and invasion in LUAD via the downregulation of miRNA-223. Zhuang et al. found that *ZNF667-AS1* and ankyrin 2 (*ANK2*) were downregulated in colorectal carcinoma [32]. Downregulated *ZNF667-AS1* expression is associated with disease progression and poor prognosis. In turn, *ZNF667-AS1* overexpression was shown to inhibit the proliferation, invasion, and migration of VOLO cells in vitro and in vivo, as well as down- and upregulated Janus kinase 2 (JAK2) and ANK2, respectively [32]. In essence, *ZNF667-AS1* interaction with ANK2/JAK2 could be an important driver of colorectal carcinogenesis. A similar study demonstrated that both *ZNF667-AS1* downregulation in the tumor tissues and low expression of *ZNF667-AS1* are associated with low overall survival rate of patients with colon cancer [33]. Moreover, the overexpression of *ZNF667-AS1* resulted in decreased invasion and migration rates of colon cancer cells, whereas treatment with transforming growth factor β1 (TGF-β1) resulted in increased invasion and migration rates of the same cancer cells. Therefore, it is postulated that *ZNF667-AS1* inhibits the invasion and migration of colon cancer cells by inactivating *TGF-β1* [33]. Another study demonstrated that *ZNF667-AS1* is downregulated in bladder cancer, whereas miR-146a-5p is upregulated. Moreover, miR-146a-5p and *ZNF667-AS1* seem to function antagonistically with overexpression of miR-146a-5p promoting the invasion, migration, and proliferation of bladder cancer cells, whereas overexpression of *ZNF667-AS1* resulted in inhibition of the migratory and proliferative capacity of the cancer cells. Overall, the study suggests that *ZNF667-AS1* may modulate the behavior of bladder cancer cells via the downregulation of miR-146a-5p [34]. Lu et al. demonstrated that *ZNF667-AS1* expression was downregulated in hepatocellular carcinoma (HCC), while the expression of *NOTCH1* was upregulated [35]. *ZNF667-AS1* and *NOTCH1* were also inversely correlated across HCC tissues. Moreover, *ZNF667-AS1* expression was lower in metastatic HCC patients than nonmetastatic HCC patients. Furthermore, *ZNF667-AS1* overexpression inhibited the migration and invasion of HCC cells, while *NOTCH1* overexpression promoted the migration and invasion of HCC cells. This suggests that *ZNF667-AS1* overexpression may inhibit HCC by downregulating *NOTCH1*.

*ZNF667-AS1* was found to be upregulated in acute myeloid leukemia (AML) patients and predicted poor prognosis [36]. Mechanistically, *ZNF667-AS1* acts as a molecular sponge for miR-206 and possibly potentiates AML progression via the targeting of the miR-206/AKAP13 axis. Yuan et al. investigated both the diagnostic value and clinical significance of the *ZNF667-AS1* expression in patients with glioma, and their findings suggest that *ZNF667-AS1* could be used as a diagnostic and prognostic biomarker in glioma patients [37].

## 4. Dysfunction of LncRNA *MORT* (*ZNF667-AS1*) in Gynecological Cancers

Several studies have addressed the role of lncRNAs in GCs [38]. The functional and possible clinical roles of *ZNF667-AS1* in GCs are reviewed (Table 2). 

### 4.1. Ovarian Cancer

Ovarian cancer (OC) is the gynecological malignancy with the poorest prognosis [44]. OC has a high mortality rate, which is attributed to the delayed onset of symptoms compounded by lack of effective screening and early detection [45,46,47,48,49]. The incidence of OC varies across the world, with the highest prevalence of 12 per 100,000 in non-Hispanic white women, followed by 10.3 per 100,000 in Hispanic, 9.4 per 100,000 in non-Hispanic black, and 9.2 per 100,000 in Asian/Pacific Islander women [50].

OC is an umbrella term for a highly heterogeneous group of tumors that are behaviorally, morphologically, and molecularly very different. Up to 90% of OCs are of epithelial origin [38]. OC is commonly associated with alterations in *TP53* and *BRCA1/2*, which are linked with an increased risk of cancer and poor prognosis [51,52,53]. In spite of the great efforts made for early detection of OC and the development of radiotherapy, novel chemotherapy, targeted therapy, and multidisciplinary treatments, there still is five-year overall survival rate of less than 50% patients [54]. Thus, the need for highly sensitive and specific diagnostic tools that identify OC at an earlier stage together with the development of new therapeutic approaches are very much needed to improve patient survival rate.

LncRNAs have been investigated in samples of patients with OC using several molecular techniques [55] and compared with samples from healthy patients or adjacent normal tissue in patients with OC. Chen et al. found that *ZNF667-AS1* is downregulated, and miRNA-21 is upregulated in tumor tissues relative to adjacent healthy tissues of patients with OC [39]. Such aberrant lncRNA expression has been shown to correlate clinicopathologically. Functionally, the overexpression of *ZNF667-AS1* has been shown to suppress the proliferation of OC cells through miRNA-21 inhibition. Although the molecular mechanism of the regulation of miRNA-21 by *ZNF667-AS1* is still not understood, this study provides new insights into the underlying pathogenesis of OC.

### 4.2. Cervical Cancer

Cervical cancer (CC) is the second leading cause of cancer-related death in women worldwide [56]. In 2018, over half a million new cases of cervical cancer were reported, with more than 300,000 deaths [56]. Clinically, CC is associated with persistent infection with ‘high-risk’ human papillomaviruses (HPVs). The subtypes HPV16 and HPV18 are responsible for the majority of precancerous cervical lesions and the development of CC [57,58]. Conventional cytology using PAP smears and liquid-based cytology are the main screening tests utilized for the detection of pre-invasive cervical disease [59]. Patients with early-stage CC can be successfully treated with radical hysterectomy without the need for adjuvant chemotherapy or radiotherapy. However, in locally advanced and advanced CC, radical surgery does not improve the chances of survival, and combinations of chemotherapy and radiotherapy are the cornerstone of treatment. The disease becomes more difficult to successfully treat as the stage of presentation advances. Although the HPV-associated carcinogenic pathway of cervical cancer is better understood than other gynecological malignancies, further in-depth studies are necessary to aid in the discovery of novel molecular therapeutic targets that would contribute to the management of patients with advanced or recurrent disease.

In CC, there is an aberrant expression of various lncRNAs such as *HOTAIR* [60], *H19* [61], *GAS5* [62], *CCAT2* [63], *ANRIL* [64], *lncRNA LET* [65], and *lncRNA-CCHE1* [66]. Li et al. showed that upregulation of *ZNF667-AS1* could lead to a reduction in the proliferative and metastatic potential of CC by modulating the miR-93-3p-dependent PEG3. This suggests that this could be a potential therapeutic target for CC treatment [40].

Epigenetic silencing of *ZNF667-AS1* in CC is correlated with decreased overall survival and increased tumor bulk [41]. Moreover, the upregulation of *ZNF667-AS1* inhibits the proliferation of CC cells. Overall, these findings suggest that *ZNF667-AS1* could be used as both a biomarker and a therapeutic target in the management of CC. Zheng et al. established an immune-related lncRNAs (IRLs) signature with a prognostic value for cases of CC [42]. The IRLs could help establish a system whereby CC patients are stratified CC into low- and high-risk groups. In fact, these IRLs, including *ZNF667-AS1* among other lncRNAs, significantly correlated with the infiltration of immune cells. These results could provide future immunotherapeutic approaches that may assist with both tumor treatment and tumor prevention.

### 4.3. Endometrial Cancer

Endometrial cancer (EC) includes endometrioid endometrial cancer (80%), clear cell cancer, and uterine serous cancer [67,68]. EC is the second most common type of gynecological tumor [69], comprising 4.8% of worldwide cancer incidence and 2.1% of mortality related to cancer [70]. EC originates in the glandular epithelial cells of the endometrium [71]. Some of the underlying risk factors include early menarche, obesity, diabetes mellitus, dietetic factors nulliparity, late menopause, advanced age, nulliparity, breast cancer tamoxifen therapy, radiotherapy, and high circulating levels of estrogen [72,73,74]. The standard treatment is total hysterectomy and bilateral salpingo-oophorectomy, which is usually effective for stage I disease [75]. In advanced stages, surgery is followed by radio- and/or chemotherapy.

There is evidence of the importance of lncRNAs and their inhibitory effects on the proliferation and metastasis of endometrial cancer cells [76,77,78]. Even though the underlying mechanisms of EC cancer initiation and development are not clear, emerging studies highlight the importance of lncRNAs in cancer etiology. Two studies by Vrba et al. [20,43] demonstrated that DNA methylation status of *ZNF667-AS1* is linked to tumor progression. Overall, these results present strong circumstantial evidence for a tumor suppressor role for *ZNF667-AS1.* This gene is among the most common epigenetic aberrations present in human cancers, including EC and uterine carcinosarcoma (UCS), which is a rare and aggressive variant of endometrial cancer [79,80,81].

## 5. Epigenetic Silencing of LncRNA *MORT* (*ZNF667-AS1*) in Cancer

The complex multifactorial nature of cancer makes diagnosis and treatment very challenging [82]. The epigenetic basis of cancer development has revolutionized cancer genetics and provided novel therapeutic targets [83,84]. Epigenetic mechanisms involve modification of chromatin structure, conferring differential gene expression without changing the DNA sequence [82]. Epimutations change the genome’s structure and stability. These have been proposed as driver mutations in the initiation of tumors and, in combination with genetic lesions, propagate carcinogenesis [82].

In cancer, epigenetic alterations include aberrant DNA methylation (hypermethylation and hypomethylation), histone modification (acetylation, methylation, and phosphorylation), and changes in gene expression by non-coding RNAs [85]. Moreover, several genes that are in charge of proliferation, stem cell differentiation, and apoptosis undergo epigenetic modifications. In particular, the aberrant DNA methylation, catalyzed by the family of DNA methyltransferases (DNMT1, DNMT3A, and DNMT3B), is commonly linked with genetic instability and carcinogenesis through the inactivation of specific cancer-related genes [82]. Specifically, the abnormal methylation of CpG dinucleotides (CpG islands), which are present in approximately 70% of human promoters, leads to direct interference with RNA polymerase II assembly and transcription factors. DNA methylation can also act as a platform for various chromatin remodeling enzymes, such as histone deacetylases (HDACs), and thus lead to chromatin condensation [86,87]. However, studies show that, during carcinogenesis, there is repression of tumor suppressor genes by hypermethylation of their promoters [88,89].

The *ZNF667-AS1* gene, as already described, was found to be a target of epigenetic silencing, specifically at the boundary where finite lifespan human mammary epithelial cells (HMEC) transition from mortal to immortal. An important change in gene expression was found during the immortalization step, which involved the downregulation of *ZNF667-AS1*, secondary to DNA hypermethylation of its CpG island promoter [20]. This was supported by the ability of the DNA methyltransferase inhibitor, 5-aza-2′-deoxycytidine, to reactivate the expression of *ZNF667-AS1*.

The DNA hypermethylation of *ZNF667-AS1* and its subsequent gene silencing, has also been identified as an early epigenetic event occurring in human carcinogenesis. This has been observed in cohorts of patients with breast ductal carcinoma in situ, suggesting an increased risk of breast cancer transformation and metastasis [90]. It was also reported when comparing colon adenoma and carcinoma cohorts, further confirming that *ZNF667-AS1* silencing is likely to occur in the premalignant state [90]. *ZNF667-AS1* is known to be frequently silenced in numerous cancers such as melanoma [30], colorectal carcinoma [32], and gastric cancer [91]. In a study characterizing the epigenetic landscape of genes encoding lncRNAs across 6475 tumors and 455 cancer cell lines, *ZNF667-AS1* was identified to be a hypermethylated lncRNA in most tumors [92]. Overall, these studies suggest that promoter hypermethylation may be one of the mechanisms in leading to *ZNF667-AS1* silencing in cancer.

## 6. Epigenetic and Gene Therapies for Gynecological Cancer Treatment

*ZNF667-AS1* expression is controlled epigenetically and the *ZNF667-AS1* loss due to aberrant DNA methylation occurs in several cancers. Several studies suggest that *ZNF667-AS1* could serve as a candidate tumor suppressor gene associated with the pathogenesis and progression of several human cancers, including GCs [20,78,90]. Thus, the expression of *ZNF667-AS1* could be restored via epigenetic modification, which may suggest therapeutic potential for the treatment of GCs.

Novel therapeutic agents using different modes of action, having synergy with current conventional management, are very much needed, especially in RGCs that carry a poor prognosis. Genetic and epigenetic factors have been shown to have a central role in the development of cancer. Gene therapy approaches have been developed, and these include suicide gene therapy, anti-angiogenic gene therapy, siRNA therapy, immunotherapy, oncolytic virotherapy, pro-apoptotic gene therapy, and gene-directed-enzyme prodrug therapy [93,94].

Epigenetic dysregulation frequently occurs in GCs. This includes histone modifications, altered methylation at CpG islands within gene promoter regions, and global demethylation leading to genome instability [95,96,97,98]. Since the epigenome can be reprogrammed, epigenetic alterations are potentially reversible and have great plasticity, unlike genetic mutations [96,97,98]. Therefore, a promising therapeutic strategy, including the provision of molecular biomarkers for diagnosis, is the possible reprogramming of the epigenome, targeting epigenetic marks and thus changing the cell landscape [83,84,86,88,99,100,101]. Since epigenetic therapies simultaneously target multiple pathways, tumors with complex mutational landscapes may be most responsive to such therapeutic approaches as they are affected by multiple driver mutations. Furthermore, some patients diagnosed with GCs such as ovarian, cervical, and endometrial cancers may have biomarkers that are potentially sensitive to epigenetic drugs (epi-drugs) [95].

However, the major challenge for epi-drugs is to translate the efficacy in vitro into effective clinical use, which is well tolerated. Several drugs have successfully reactivated otherwise silenced tumor suppressor genes in several cancer cell lines, but failed to achieve a significant response in clinical trials [87,88,102]. Currently, some commonly used epi-drugs, used alone or in combination with other anti-cancer agents, lack specificity and have generated unwanted epigenetic modifications [103,104]. Another stumbling block is the acquired resistance to certain epi-drugs. In the era of personalized medicine, high-throughput mapping technologies allow the genome and epigenome mapping of a specific cell population from an individual patient. This facilitates testing for drug sensitivity, enabling an efficient and specific patient-tailored treatment approach [105].

GCs can also benefit greatly from targeted gene delivery therapies. Gene therapy usually involves directly injecting a gene, or vectors to deliver a gene, into the cells where that specific gene is required. The cell’s gene-reading machinery uses the information in this injected gene to produce RNA and protein molecules [106,107]. The development of safe vectors and optimal delivery to the region of interest determines the successful outcome of this therapy. Several studies have indicated that transcriptional regulatory sequences of the *H19* gene are potentially important candidates for cancer gene therapy [108,109,110,111]. *H19* is an oncofetal gene that is paternally imprinted and maternally expressed and that encodes a lncRNA. The key feature of *H19* is that it is expressed in many human tumor types, but there is little to no expression in normal adult tissues. This suggests a role for *H19* in promoting angiogenesis, cancer progression, and metastasis, and targeting the *H19* promoter region might be used in gene therapy [112]. This enables the directing of a tumor-selective promoter in conjunction with a cytotoxic gene. The Luc-H19 plasmid carrying a diphtheria toxin subunit regulated by the H19 promoter is effective in early phase studies against several ovarian cancer cell lines and primary cells [109]. The injection of this plasmid into tumor cells can lead to a reduction in tumor size due to the high levels of diphtheria toxin that are produced. Some lncRNAs (e.g., *PTENP1*, *MEG3*, or *ZNF667-AS1*) are downregulated in cancer samples when compared to normal tissues. Thus, in these cases, cancer cells could be treated with the delivery of tumor suppressor RNAs, resulting in the increased expression of the downregulated lncRNAs with subsequent reduction in tumor size and proliferative capacity [112]. However, lncRNA overexpression is usually technically challenging. This is because lncRNA overexpression usually requires vectors and delivery systems able to carry long transgenes with high efficiency of transfection. One possible alternative to circumvent the drawbacks of cell transfection in vivo is to transfect target cells in vitro, which are then transplanted for therapy. In fact, this is carried out in many of the in vivo studies exploring the biological role of lncRNAs [113].

## 7. Conclusions and Future Directions

**Figure 2:**
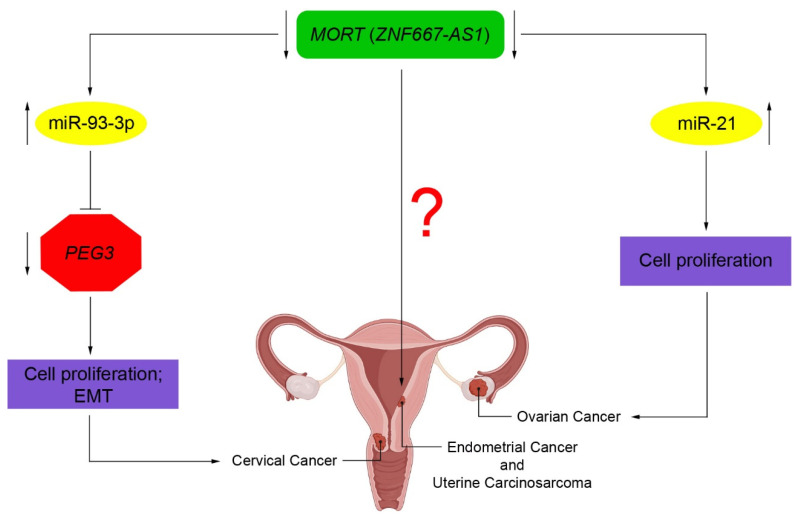
Pathological role of *MORT* (*ZNF667-AS1*) in gynecological cancers. Underexpression of *MORT* (*ZNF667-AS1*) could promote tumor initiation in ovarian, cervical, and endometrial cancers through different molecular mechanisms not yet well explored. In ovarian cancer, *MORT* (*ZNF667-AS1*) downregulation and consequent miR-21 overexpression promote the proliferation of ovarian cancer cells. In cervical cancer, downregulation of *MORT* (*ZNF667-AS1*) and consequent overexpression of miR-93-3p reduce the expression of *PEG3*, thereby allowing cell proliferation and EMT. In endometrial cancer and uterine carcinosarcoma, *MORT* (*ZNF667-AS1*) expression is silenced by aberrant DNA methylation. However, its function mechanism in these types of cancer is unknown. BioRender has been used to create parts of this figure. (https://biorender.com, accessed on 18 May 2021).

GCs continue to have a major impact on the female population. Despite vast improvements in clinical care, survival rates continue to be low for many of these cancers. The development of novel therapies for GCs is of high priority to improve the chances of earlier diagnosis and overall survival. There is a great potential in using lncRNAs for cancer therapy [114]. Despite the increasing amount of research involving lncRNAs, the understanding of their role in GCs is still in its infancy. The dysregulation of many lncRNAs has been linked with clinical features, and this could prove useful for the future development of novel biomarkers for both diagnosis and prognosis. Overall, the studies outlined suggest that *ZNF667-AS1* acts as a tumor suppressor in several types of cancer, including GCs (Figure 2) and might be considered as a potential diagnostic marker. It may even prove to be an effective therapeutic target for GCs, including “rare” forms. However, more clinical studies are required to determine the diagnostic and prognostic potential of *ZNF667-AS1* in GCs. A deeper understanding of epigenetic changes, particularly involving lncRNAs, are likely to enhance the efficiency of the application of lncRNAs as potential clinical biomarkers and therapeutic targets in cancer management.

## Figures and Tables

**Table 1:** Pathological role of *MORT (ZNF667-AS1)* in cancer.

Cancer Type	Role	Mechanism	Effect	Reference
LSCC	Tumor suppressor	Upregulates ZNF667 expression	Reduces proliferation, migration, and invasion	[26]
OSCC	Tumor suppressor	Downregulates ROCK1 expression	Reduces proliferation	[27]
NPC	Tumor suppressor	Sponge for miR-1290	Reduces proliferation both in vitro and in vivo	[28]
ESCC	Tumor suppressor	Upregulates ZNF667 expression	Reduces viability, migration, and invasion	[29]
UM	Tumor suppressor	Upregulates MEGF10 expression	Inhibits cell proliferation as well as induces apoptosis and cell cycle arrest	[30]
LUAD	Tumor suppressor	Downregulates miR-223	Inhibits cancer cell invasion and migration	[31]
CRC	Tumor suppressor	Regulates ANK2/JAK2 expression; Regulates TGF-β1 expression	Inhibits proliferation, invasion and migration; Reduces migration and invasion	[32,33]
Bladder	Tumor suppressor	Downregulates miR-146a-5p	Inhibits proliferation, invasion and migration	[34]
HCC	Tumor suppressor	Downregulates NOTCH1 expression	Inhibits the migration and invasion	[35]
AML	Oncogene	Sponge for miR-206	Enhances cell proliferation, invasion, tumorigenesis, and metastasis	[36]
Glioma	Oncogene	Unknown	Enhances cell proliferation	[37]

Abbreviations. LSCC: laryngeal squamous cell carcinoma; OSCC: oral squamous cell carcinoma; NPC: nasopharyngeal carcinoma; ESCC: esophageal squamous cell carcinoma; UM: uveal melanoma; LUAD: lung adenocarcinoma; CRC: colorectal carcinoma; HCC: hepatocellular carcinoma; AML: acute myeloid leukemia.

**Table 2:** Expression of *MORT (ZNF667-AS1)* and its association with clinicopathological features in gynecological cancers.

Cancer Type	Up-/Downregulated or Silenced	Related mRNA/miRNA	Effects	Sources	Clinico- Pathological and Prognostic Significance	Ref.
Ovarian cancer	Down	miR-21	*MORT (ZNF667-AS1)* inhibits cell proliferation by miR-21 inhibition	72 tumor tissues and adjacent healthy tissues; UWB1.289 and UWB1.289+BRCA cells	*MORT (ZNF667-AS1)* expression is affected by tumor size, but not by tumor metastasis	[39]
Cervical cancer	Down	miR-93-3p, PEG3	*MORT (ZNF667-AS1)* suppresses proliferation and metastasis via the modulation of miR-93-3p-dependent PEG3	GEO datasets; 64 cancer tissues and adjacent normal tissues; HeLa and C-33A cells	Unknown	[40]
	Down	Unknown	*MORT (ZNF667-AS1)* inhibits cell proliferation	GEO and TCGA datasets; 60 cancer tissues and 30 normal tissues; HeLa and SiHa cells, and HcerEpic (used as control cell line)	Low levels of *MORT (ZNF667-AS1)* are correlated with decreased overall survival as well as increased tumor size	[41]
	Down	Unknown	Unknown	GSE6791 and TCGA datasets	*MORT (ZNF667-AS1)* could stratify CC patients into the low- and high-risk groups	[42]
Endometrial cancer (EC)	Silenced by aberrant DNA methylation	Unknown	Unknown	TCGA datasets	Unknown	[20]
Uterine carcinosarcoma (UCS)	Silenced by aberrant DNA methylation	Unknown	Unknown	TCGA datasets	Unknown	[43]

## Data Availability

This review paper does not report any new data.

## Abbreviations

AML	acute myeloid leukemia
CC	cervical cancer
CRC	colorectal carcinoma
EC	endometrial cancer
ESCC	esophageal squamous cell carcinoma
HCC	hepatocellular carcinoma
HPV	human papillomavirus
IRLs	immune-related lncRNAs
LncRNA	long non-coding RNA
LSCC	laryngeal squamous cell carcinoma
LUAD	lung adenocarcinoma
miRNA/miR	micro-RNA
MORT	mortal obligate RNA transcript
ncRNA	non-coding RNA
NPC	nasopharyngeal carcinoma
OC	ovarian cancer
OSCC	oral squamous cell carcinoma
PAP	Papanicolaou
RGCs	rare gynecologic cancers
UCS	uterine carcinosarcoma
UM	uveal melanoma
ZNF667-AS1	ZNF667 antisense RNA 1 (head to head)
